# Optimizing sampling efforts for ex situ conservation of genetic variability of *Dipteryx alata* Vogel

**DOI:** 10.1186/1753-6561-5-S7-P18

**Published:** 2011-09-13

**Authors:** Dayane B Melo, José Alexandre F Diniz-Filho, Guilherme Oliveira, Ludmilla L Santana, Thannya N Soares, Lázaro J Chaves, Ronaldo V Naves, Rosane G Collevatti, Mariana P de C Telles

**Affiliations:** 1 Programa de Pós-Graduação em Agronomia (UFG), Brazil; 2 Depto. Ecologia, Universidade Federal de Goiás (UFG), Brazil; 3 Programa de Pós-Graduação em Ecologia & Evolução, ICB, UFG, Brazil; 4 Laboratório de Genética & Biodiversidade, Depto. Biologia Geral, ICB, UFG, Brazil; 5 Escola de Agronomia & Engenharia de Alimentos, UFG, Brazil

## Background

The “Baru” tree (*Dipteryx alata* Vogel) is an endemic species from the Cerrado biome, but widely distributed within the biome and abundant in several of its habitats [1].The species is also important in commercial terms for recover of degraded areas, and seeds are consumed *in natura* and are source of raw material for small and middle-sized food industries [2].Thus, a more detailed knowledge of its population variability and structure is needed for better establishing both *in situ* and *ex situ* conservation efforts. Data for such analyses can be provided by new molecular markers, such as microsatellites, whereas spatial analyses and optimization procedures can be applied to analyze these data and thus improve conservation efforts. Here we investigate the genetic variability within the germplasm bank of *D. alata* based on microsatellite data and compared it with a large sample of 25 widely distributed natural populations of the species. We then used a simulated annealing algorithm to find the smallest number of these natural populations that should be sampled to conserve the full genetic variability of the species.

## Methods

We described the genetic variability of 816 individuals of *D. alata* from 25 natural population widely distributed in the Cerrado biome, and from 180 individuals preserved in an active germoplasm bank, situated in the “Escola de Agronomia e Engenharia de Alimentos” Universidade Federal de Goiás, totalizing 996 individual plants genotyped for 9 microsatellite loci. The origin of the individuals in the bank is not known in detail, but it is certain that they came from several localities from Goiás State.

Overall description of genetic variability in the natural populations and in the germoplasm bank was done by percentage of polymorphic loci (P), mean number of alleles per loci (A), expected heterozigosity under H-W equilibrium (He) and observed heterosigozity (Ho). Population structure and divergence among natural populations were analyzed using *F_ST_*statistics.

A simulated annealing algorithm, implemented in the software SITES [3] was used to establish the minimum number of local populations necessary to complement the germoplasm bank so that all alleles (expressed as present or absent in each local population) are represented at least once (i.e., representation goal). The search was performed using two hundred runs and 10,000,000 iterations for each run. The problem is analogous to the ecological problem of finding a minimum number of new conservation priority areas that represent all species, after fixing a few already established natural reserves (see [4]).However, there may be frequently multiple combinations of local populations that satisfy the representation goal, so the first 100 solutions of SITES were also retained. The relative frequency of each local population in the alternative optimized solutions is an estimate of the “irreplaceability”, or relative importance, of the populations to the overall goal of representing the entire genetic variablity (alleles).

## Results and discussion

The genetic variability of *D. alata* for the microsatellite loci analyzed here was low compared with other forest species in the same region (e.g., [5]). Out of the 9 loci, on average 78% were polymorphic within natural population, and 67% were polymorphic in the germplasm bank. The mean number of alleles per loci were 2.7 and 4.4 for the natural populations and for the germplasm bank, respectively. The *He* and *Ho* were equal to 0.36 and 0.30 for natural populations, and 0.36 and 0.23 for the germoplasm bank. The F_ST_ was significant and equal to 0.259, indicating a relatively high differentiation among the local populations.

**Figure 1:**
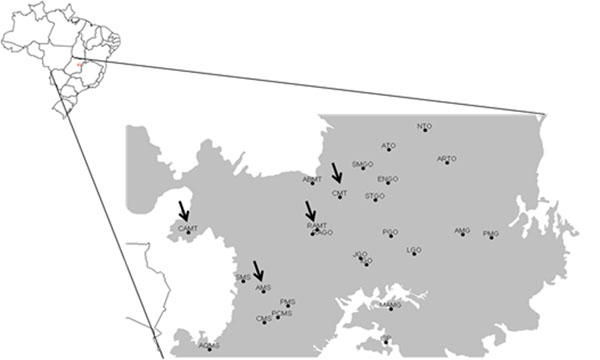
Natural populations (red arrows) of *D. alata* that must be sampled to achieve the conservation goal of representing all alleles from 9 microsatellite loci complementing, in an optimum way, the genetic variability found in the germoplasm bank reared at “Escola de Agronomia, Universidade Federal de Goiás”*.*

The simulated annealing revealed that only 4 local populations, located in Mato Grosso and Mato Grosso do Sul (Figure 1; 1-CMT. 5-AMS. 19-RAMT e 25-CAMT), are necessary to represent all alleles that appeared in the species, complementing in an optimum way the genetic variability already present in the germplasm bank. The solution is unique so that all these 4 local populations have 100% of irreplaceability.

Our analyses confirm that the germoplasm bank currently established in the Universidade Federal de Goiás preserves the genetic variability of a relatively large proportion of species’ range in the Cerrado biome. To improve such representation it is important, however, to sample more local populations in the west portion of the species’ distribution, in Mato Grosso region. It is important to note that these populations possess unique alleles that are not found elsewhere, so they are irreplaceable for achieving the conservation goal. If these populations are lost (i.e., extinct), there will be genetic erosion reducing total genetic variability in this species. So, an effort to sample and grow up individuals or progenies from the 4 populations, preferentially coupled with in situ conservation programs in these natural populations from Mato Grosso region, must be urgently done. Our optimization analyses reveals that only a few samples are necessary to achieve a more complete representation for the species, and this is important to reduce sampling and maintenance costs without losing efficiency in terms of establishing conservation and future breeding programs for the species.
